# Modeling the time-dependent transmission rate using gaussian pulses for analyzing the COVID-19 outbreaks in the world

**DOI:** 10.1038/s41598-023-31714-5

**Published:** 2023-03-18

**Authors:** Setianto Setianto, Darmawan Hidayat

**Affiliations:** 1 Department of Physics, FMIPA, Universitas Padjadjaran, Jalan Raya Bandung-Sumedang KM 21, Sumedang, 45363 Indonesia; 2 Department of Electrical Engineering, FMIPA, Universitas Padjadjaran, Jalan Raya Bandung-Sumedang KM 21, Sumedang, 45363 Indonesia

**Keywords:** Computational biology and bioinformatics, Diseases

## Abstract

In this work, an SEIR epidemic model with time-dependent transmission rate parameters for the multiple waves of COVID-19 infection was investigated. It is assumed that the transmission rate is determined by the superposition of the Gaussian pulses. The interaction of these dynamics is represented by recursive equations. Analysis of the overall dynamics of disease spread is determined by the effective reproduction number *R*_*e*_(*t*) produced throughout the infection period. The study managed to show the evolution of the epidemic over time and provided important information about the occurrence of multiple waves of COVID-19 infection in the world and Indonesia.

## Introduction

The use of epidemic models makes it possible to simulate the dynamics of disease transmission to detect emerging outbreaks and assess public health interventions^1–5^. A standard method, SEIR (susceptible-exposed-infectious-removed), was developed to describe the epidemic dynamics, which takes into account the time-dependent coefficients and offers the possibility to analyze the epidemic dynamics^6–8^. The method was applied to the 2019 coronavirus (COVID-19) pandemic caused by the severe acute respiratory syndrome Coronavirus-2 named SARS-CoV-2^3,4,9–11^. In addition, this method allows us to evaluate trends and forecasts in the number of cases of infection in order to assess possible influencing factors such as the availability of vaccines^12,13^ or restrictive measures by central authorities^14,15^ may affect the outbreak of the epidemic. The SEIR model with time-varying parameter specifications has been used in several studies known in the literature^16–20^. However, they cannot consider external influences, such as actions that contain the spread of an infection that may occur at different times during the development of the infection itself or possible changes in the health condition of the infected individual due to pharmacological development.

Therefore, we describe a research work focused on using Gaussian pulses to model the time-dependent transmission rate of COVID-19. The Gaussian pulses are used to capture the dynamic nature of the transmission rate, which is subject to change as a result of various factors such as government interventions, vaccination campaigns, and changes in human behavior. The study aims to improve the understanding of the spread of COVID-19 by using an SEIR epidemic model with time-dependent transmission rate parameters to analyze the multiple waves of COVID-19 infection. By studying the epidemic dynamics and analyzing the effective reproduction number *Re*(t) throughout the infection period, the study can provide insights into how the virus spreads. This information can be used to develop more accurate and detailed strategies for controlling and preventing the spread of COVID-19.

As an implementation and test model, we apply it to the situation of the spread of COVID-19 in the real world and in Indonesia by using data from sources^21^ with the quality of the observed data and its validation is an important conclusion in assessing the reliability of the results of this model. The term "world" in the title is used to indicate that the data or information being referred to is not limited to a specific region or country, but rather encompasses a “global” perspective. Additional information from the results of our model is to know the overall dynamics of the spread of the COVID-19 disease in the world which is determined by the effective reproduction number produced during the entire infection period. So that the spread of the disease can be tracked and used as crucial data for decision-makers and the public to take action in anticipation of the next surge of COVID-19 cases.

## Methodology

### Database

The analysis is based on data collected (daily and total cases) from the website (https://www.worldometers.info/about/) run by an international team of developers, researchers, and volunteers to collect various statistical data from around the world real to represent time^22^. For the COVID-19 pandemic, they manually analyze, validate and collect real-time data from thousands of sources around the world. By definition, the total number of cases is the cumulative total number of reported cases that were found to be clinically confirmed (criterion by country). Active Cases = (Total Cases) − (Total Deaths) − (Recovered). This is data on the number of people currently infected with the virus. We will implement our model using the world population data of 7,794,798,739 on July 1, 2020, and 273,523,615 for Indonesia population^23^.

### Modified seir epidemic model

We started by introducing the SEIR model, which is one of the most widely used extensions of the standard SIR model, the epidemiological model based on Ordinary Differential Equations (ODE). In general, the SEIR models classify the population into four classes: Susceptible (S), Exposed (E), Infected (I), and Recovered or Removed (R). The model describes how different parts of the population change over time. The total population is *N* = *S* + *E* + *I* + *R*. The interaction of these dynamics is represented by recursive formulation^24,25^:

1 $$S_{t + 1} = S_{t} - \left( {\frac{{r\beta S_{t} I_{t} }}{N}} \right)\Delta t$$

2 $$E_{t + 1} = E_{t} + \left( {\frac{{r\beta S_{t} I_{t} }}{N} - \sigma E_{t} } \right)\Delta t$$

3 $$I_{t + 1} = I_{t} + \left( {\sigma E_{t} - \gamma I_{t} } \right)\Delta t$$

4 $$R_{t + 1} = R_{t} + \gamma I_{t}\Delta t$$

These four equations are called Euler formulas of an existing SEIR model and can be employed to generate a set of values that reflect what is happening with the epidemic over a period of time. Where *r* is the number of contacts per unit of time, *β* is the probability of disease transmission per contact which is the transmission rate, *σ* is a per-capita rate of progression to infectious state which is the loss of latency rate, and *γ* is the per-capita recovery rate. This formulation assumes full population composition rather than population dynamics (i.e. natural births and deaths are considered negligible during epidemic periods)^13^. In order to calculate anything from these formulas we need to have explicit values for *β*, *σ*, *γ*, *S*, *E*, *I*, and *R*. In the standard SEIR model the parameters *β*, *σ*, and *γ* is a constant that does not depend on time. However, the characteristics of the epidemic show us that these parameters can change and vary over time. Note that in some models for diseases where contacts are not well defined (e.g. influenza and COVID-19), *rβ* (the number of contacts per unit time multiplied by the probability of disease transmission per contact) are combined into one parameter (often also referred to as the constant of *β*, the number of adequate contacts per unit of time)^26,27^. For this reason, we modify the SEIR model by proposing to express the number of suitable contacts per unit of time *β* is expressed as a function of time, the multiple Gaussian pulse^28^. which combines the m-Gaussian function as defined:

5 $$\beta \left( t \right) = \mathop \sum \limits_{m = 1}^{M} r_{m} e^{{ - \frac{{\left( {t - t_{m} } \right)^{2} }}{{d_{m}^{2} }}}}$$

where *r*_*m*_, *t*_*m*_, and *d*_*m*_ are amplitude (number of contacts), the center, and half of the pulse duration respectively with *m* = 1, 2, 3, …, *M*. This model can be referred to as a one-dimensional Gaussian Mixture Model (GMM)^29,30^. Using multiple Gaussian pulses allows greater flexibility in designing the transmission rate model and can provide a more accurate representation of a data set^31^. This is because the various parameters of each Gaussian pulse can be adjusted to produce a desired effect or response. So, Eqs. (1) and (2) are simplified as:

6 $$S_{t + 1} = S_{t} - \left( {\frac{{\beta_{t} S_{t} I_{t} }}{N}} \right)\Delta t$$

7 $$E_{t + 1} = E_{t} + \left( {\frac{{\beta_{t} S_{t} I_{t} }}{N} - \sigma E_{t} } \right)\Delta t$$

The transmission rate of COVID-19, also known as the basic reproduction number (*R*_0_), is an estimate of how many people will be infected by one infected person in a population where everyone is susceptible to the disease. *R*_0_ is an uncertain parameter in the SEIR model because it can be difficult to accurately measure in real-world settings^32^. In fact, the average number of new infections caused by a single infected person at the time of the partially susceptible population can be determined by the effective reproduction number *Re* generated throughout the infection period, which varies proportionally to the population of the susceptible population over time be changed:

8 $$R_{e} \left( t \right) = \frac{{\beta _{t} }}{\gamma }\frac{{S\left( t \right)}}{N}$$

The effective reproduction number indicates the potential rate of spread of the disease in a population. When *R*_*e*_ is greater than 1, it suggests that the disease is spreading, while when *R*_*e*_ is less than 1, it suggests that the disease may be slowing down. When *R*_*e*_ is equal to 1, it indicates that the number of new infections is stable, but not necessarily that the infected population is in equilibrium^19,33–36^.

### Parameter estimation of the model

Parameter estimation is the process of estimating the values of unknown parameters in a mathematical model based on observed data. In the context of epidemic modeling, parameter estimation involves estimating the values of parameters that govern the transmission dynamics of the disease, such as the transmission rate, the incubation period (σ), the recovery time (γ), and the effective reproduction number (*R*_*e*_). The process of parameter estimation typically involves fitting the model to observed data by adjusting the values of the unknown parameters to minimize the difference between the predicted values and the observed data. This is often done using optimization techniques such as maximum likelihood estimation, least squares regression, or Bayesian inference^37^. Parameter estimation is a critical step in epidemic modeling, as the accuracy of the model predictions depends on the accuracy of the estimated parameters. Accurate estimates of the parameters can help inform public health decisions and guide interventions to control the spread of the disease. To perform parameter estimation of the SEIR epidemic model using daily cases data, we follow the steps bellow:

- **Step 1: Define the modified SEIR model.**
  Write the equations that describe the dynamics of the SEIR model, which includes the four compartments (Eq. 1 to Eq. 7).
- **Step 2: Collect daily case data.**
  Collect information over time on the amount of daily reported cases. This data can be obtained from the website (https://www.worldometers.info/coronavirus/#countries).
- **Step 3: Define the objective function.**
  Describe the goal-oriented task as the *R*^2^ value or coefficient of determination between the observed and predicted daily case counts. The *R*^2^ value measures the proportion of variance in the observed daily case counts that is explained by the predicted values. The formula for *R*^2^ is:
  $$R^{2} = 1 - \left( {SS_{res} /SS_{tot} } \right)$$
  where *SS*_*res*_ is the sum of squared residuals between the observed and predicted daily case counts, and *SS*_*tot*_ is the total sum of squares^38^.
- **Step 4: Set up the optimization problem.**
  Create the optimization challenging problem by defining the objective function and the constraints on the parameters. The constraints may include upper and lower bounds on the parameter values or other restrictions based on prior knowledge or assumptions.
- **Step 5: Solve the optimization problem.**
  Use the Generalized Reduced Gradient(GRG) algorithm in Microsoft Excel's Solver add-in to solve the optimization problem and estimate the values of the parameters that maximize the *R*^2^ value^39,40^.
- **Step 6: Validate the model.**
  Test the SEIR model's accuracy by comparing the model predictions with the observed data. This can involve calculating the *R*^2^ value or other metrics of model fit.
- **Step 7: Use the model for the determination of (***R*_*e*_**)** **in Eq.** 8.
  Once the model has been validated, Public health officials and epidemiologists can use the Effective reproduction number *R*_*e*_ to monitor the spread of infectious diseases and to inform decisions about public health interventions, such as vaccination campaigns, quarantine measures, and social distancing policies.

In the modified SEIR epidemic model used in the study, the convergence parameter is set to the default value of 0.0001, which means that the model will stop iterating when the maximum change in the solution is smaller than this value. This ensures that the calculations have converged and the solution is accurate. However, the accuracy of the solution can also be controlled by checking the coefficient of determination *R*^2^, which is a statistical measure that indicates how well the model fits the data. In addition to accuracy, the stability of the model is also important. This can be ensured by selecting reasonable parameter values, such as the incubation period and recovery time. These parameters determine how long it takes for an individual to become infectious and how long it takes for them to recover. By selecting appropriate values for these parameters, the model can accurately simulate the epidemic dynamics and provide reliable predictions. Overall, controlling the accuracy and stability of the modified SEIR model is crucial for understanding the dynamics of disease spread and developing effective control and prevention strategies.

## Results and discussion

### Multiple waves of infection of COVID-19 disease

The COVID-19 epidemic is still present in almost all nations as of right now. As of February 10, 2023, 755,385,709 confirmed cases of COVID-19 have been reported to WHO, including 6,833,388 deaths^41^. Although the spread of COVID-19 in the world has decreased relatively in 2023, more new cases are surging and could potentially cause the spread of infections in the next few waves. These waves are caused by changes in the transmission rate due to various factors, such as changes in government policies, changes in behavior, or the emergence of new variants of the virus. We use data on COVID-19 cases from around the world (January 22, 2020, to February 13, 2023) and Indonesia from (February 15, 2022, to February 11, 2023) to implement the proposed model. Table 1. shows the best parameters from simulation results using multi-wave infection models for the global case, and Table 2. shows the best parameters from a particular case in Indonesia. According to the two tables, the fifteen Gaussian pulse models have a correlation value of *R*^2^ of 0.9520 with the COVID-19 global case data. Meanwhile, for the COVID-19 instance in Indonesia, nine Gaussian pulses with an *R*^2^ of 0.9573 were used. These values are obtained by estimating the best results from simulations obtained by non-linear regression methods compared to globally observed daily data of COVID-19 with the highest accuracy of the model determined by the coefficient of determination or correlation index (*R*^2^). In addition, the parameters of the incubation period (σ) and recovery time (γ) are also used as a consideration for selecting the model, the complete results of which are shown in Table S1 and S2 in the supplementary information file. These parameters are being used to investigate the effective reproduction number of COVID-19 infections over more than a year.

**Table 1:** Parameter estimation of multiple waves model of COVID-19 for the world (observed data from 22 January 2020 to 13 February 2023).

The world	m_1_	m_2_	m_3_	m_4_	m_5_	m_6_	m_7_	m_8_	m_9_	m_10_	m_11_	m_12_	m_13_	m_14_	m_15_	
N (July 1, 2020)																7,794,798,739
I_0_ (initial number of infected)																970
*γ* (per day)																0.0171
*σ* (per day)																0.0366
r_m_ (number of contact per day)	0.01	0.28	0.19	0.25	0.04	0.03	0.03	0.02	0.02	0.06	0.01	0.01	0.02	0.01	0.02	
d_m_ (days)	12	18	49	23	54	89	39	50	93	19	14	9	45	29	42	
t_m_ (day)	0	12	44	49	160	299	457	565	718	723	787	846	916	989	1061	
*R*^2^ (coef. of determination)																0.9520

**Table 2:** Parameter estimation of multiple waves model of COVID-19 for Indonesia (observed data from 15 February 2020 to 11 February 2023).

Indonesia	m_1_	m_2_	m_3_	m_4_	m_5_	m_6_	m_7_	m_8_	m_9_	
N (July 1, 2020)										273,523,615
I_0_ (initial number of infected)										1
*γ* (per day)										0.0696
*σ* (per day)										0.6274
r_m_ (number of contact per day)	0.14	0.05	0.13	0.09	0.03	0.1	0.25	0.1	0.1	
d_m_ (days)	12	82	22	486	32	32	27	57	30	
t_m_ (day)	1	3	40	76	328	498	710	880	994	
*R*^2^ (coef. of determination)										0.9573

#### Incubation period

The incubation period of COVID-19 is the time between exposure to the virus and the onset of symptoms. According to the World Health Organization (WHO), the incubation period for COVID-19 is typically 5–6 days, with a range of 1–14 days^38^. This means that the majority of people will acquire symptoms within 5–6 days of being infected with the virus, but some people may take up to 14 days or longer. According to this model, the incubation period of COVID-19 in Indonesia is approximately 2 days, while the global average is 27 days. It is essential to note, however, that the incubation period varies from person to person and may be affected by factors such as age, health status, and viral load of the infected person^42^.

#### Recovery time

**Figure 1:**
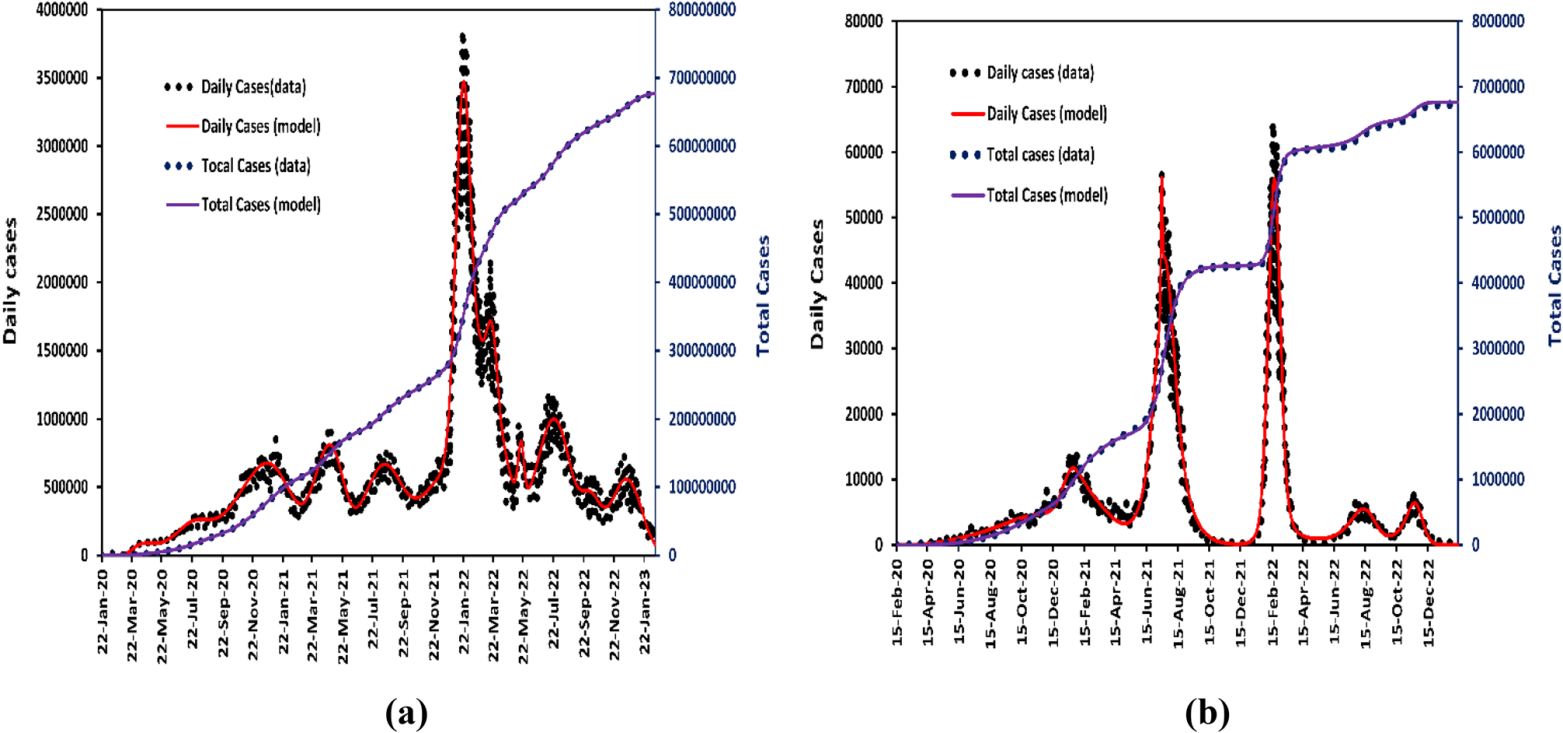
The model of multiple waves infection of the total cases (purple line), daily cases (red line), and compared with the observed data of total cases (blue dot) and daily cases (black dot) of COVID-19 for (**a**) the world from 22 January 2020 to 13 February 2023 and (**b**) Indonesia from 15 February 2020 to 11 February 2023.

The recovery time for COVID-19 can vary depending on the individual and the severity of their illness. For most people, the recovery period is typically a few weeks. For mild cases, symptoms may resolve within a few days or up to 2 weeks. For moderate to severe cases, recovery can take several weeks to a month or more. Some people may experience longer-term effects such as fatigue, shortness of breath, or other symptoms even after they have recovered. The Indonesian Ministry of Health recommends a 10-day quarantine period for people who have tested positive for COVID-19 or have been in close contact with someone who has tested positive^43^. According to this model, the recovery period for COVID-19 in Indonesia is 14 days, while the global average is 58. In general, most people with mild to moderate symptoms of COVID-19 can recover within two to six weeks, while those with severe illness may take several weeks to recover^44,45^. Considering that the incubation period and recovery time for COVID-19 have a wide range due to the many influencing factors, this parameter has a high level of sensitivity as shown in our model optimization process for changes in the number of Gaussian pulses applied. (Table S1 and S2 in the supplementary information file). Therefore, we successfully developed the multi-wave model to simulate the spread of infection due to an infectious disease. Figure 1a,b compare recent COVID-19 cases (daily and total cases) from around the world and Indonesia using our multi-wave model with m-Gaussian pulses as the transmission rate. These results convincingly explain why the approximation of the transmission rate function as multiple Gaussian pulses shows good model accuracy over the observed data.

### The effective reproduction number of COVID-19 outbreaks

**Figure 2:**
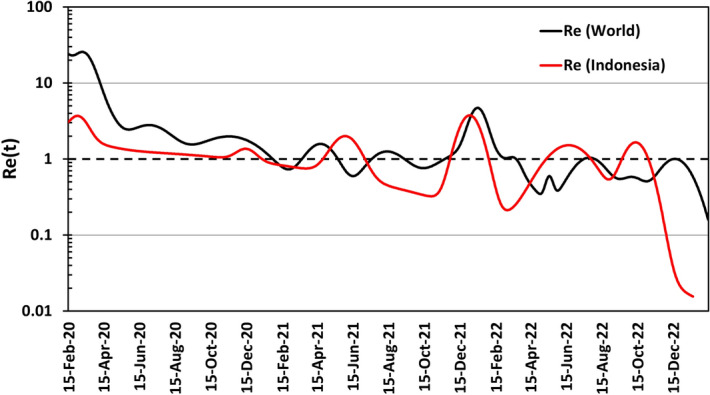
The effective reproduction number of COVID-19 for the world (black line) and Indonesia (red line).

In general, when the Covid-19 pandemic first began, the virus was spreading rapidly and there were many new cases each day. This is because each infected person was transmitting the virus to more than one other person, on average. The value of *R*_*e*_ is a measure of this transmission rate, and when it is greater than one, the number of cases is expected to increase over time^44^. As public health measures such as social distancing, wearing masks, and vaccination were implemented, the rate of transmission began to decrease^46^. This means that each infected person was transmitting the virus to fewer people, on average. As a result, the number of new cases began to decline. However, there were still times when the number of cases increased again, resulting in peaks in the number of Covid-19 infections which is then called multi waves of infection. Figure 2 shows the effective reproduction number (*R*_*e*_), which is an essential parameter in the overall dynamics of the spread of COVID-19 infection globally and in Indonesia (black and red lines). This curve clearly shows that there have been six waves of COVID-19 disease infection globally, with peaks in January 2021, May 2021, September 2021, March 2022, August 2022, and December 2022. Five infection peaks for the COVID-19 virus in Indonesia occurred in February 2021, July 2021, February 2022, August 2022, and November 2022.

During each of these peaks, the rate of transmission eventually decreased and reached an equilibrium equal to one. Since *R*_*e*_ is equal to one, it means that each infected person is transmitting the virus to exactly one other person, on average. This is because, for every infected person, there is only one other person who will become infected. When the value of *R*_*e*_ is equal to one, the number of cases is expected to remain stable over time, because each new case is offset by one recovery or removal. So, in summary, we find that this model provides a reasonable and realistic estimate of the effective reproduction number by giving information on the number of secondary infections expected from infected people over time, assuming that the transmission rate parameter uses multiple Gaussian pulses. It should be noted that the *R*_*e*_ values must be interpreted with caution since they may be affected by factors such as reporting and testing delays, which can cause a lag in determining the actual number of cases^33,47^.

## Conclusion

The article proposes a model to estimate the transmission rate of COVID-19 using Gaussian pulses. The model is based on the SEIR epidemic model, a standard mathematical model for studying the spread of infectious diseases. The advantage of using Gaussian pulses is that they can account for the phenomenon of multiple waves of infection seen in the spread of COVID-19. The analysis showed that the model successfully captures the general trends in the spread of the disease and provides insights into the incubation period and recovery time. The effective reproduction number (*R*_*e*_) is well determined by simulation results. Accurately estimating *R*_*e*_ is an important benefit as it allows for a more accurate understanding of the dynamics of the spread of the COVID-19 virus, allowing changes in its transmission rate to be tracked over time. Overall, the proposed model provides a useful tool to help public health officials and policymakers make informed decisions about interventions to limit the spread of the disease, such as social distancing measures or vaccination campaigns.

## Supplementary Information

Supplementary Tables.

## Data Availability

The datasets generated and/or analyzed during the current study are available at https://github.com/setiantos/Covid19 and its supplementary information file.
