# Supplementary material for: Modeling the time-dependent transmission rate using gaussian pulses for analyzing the COVID-19 outbreaks in the world
Source: Sci Rep. 2023 Mar 18;13:4466. doi: 10.1038/s41598-023-31714-5 (PMC10024739; doi:10.1038/s41598-023-31714-5)
Supplement: Supplementary file 1 — Supplementary Tables. [file 41598_2023_31714_MOESM1_ESM.pdf]

Supplementary Information

Modeling the Time-Dependent Transmission Rate Using Gaussian Pulses for Analyzing the COVID-19 Outbreaks in the World

Setianto Setianto[1*] and Darmawan Hidayat[2]
[1]Department of Physics, FMIPA, Universitas Padjadjaran
[2]Department of Electrical Engineering, FMIPA, Universitas Padjadjaran
Jalan Raya Bandung-Sumedang KM 21, Sumedang 45363, Indonesia
[*]corresponding author: setianto@phys.unpad.ac.id

Table S1. Parameter estimation of the modified SEIR epidemic model for the world with total cases data of COVID-19 677,732,420 on February 13, 2023

Parameter	Beta (m = 10)	Beta (m = 12)	Beta (m = 15)	Beta (m = 18)
R^2 (coef. of determination)	0.9482	0.9477	0.9520	0.9536
γ(recovery time in per days)	1/12.69	1/39.68	1/58.47	1/5000
σ(incubation period in per days)	1/10.51	1/37.03	1/27.32	1/58.82
Δ Total cases (data – model)	(2,543,228)	(-378,735)	(154,078)	(-23,994)

This supplemental material has been provided by the authors to give readers additional information about their work.

Table S2. Parameter estimation of the modified SEIR epidemic model for Indonesia with total cases data of COVID-19 6,732,618 on February 11, 2023

Parameter	Beta (m = 6)	Beta (m = 9)	Beta (m = 12)	Beta (m = 15)
R^2 (coef. of determination)	0.9539	0.9619	0.9567	0.9532
γ(recovery time in per days)	1/10.44	1/14.36	1/20.16	1/19.19
σ(incubation period in per days)	1/3.45	1/1.59	1/1.57	1/1.87
Δ Total cases (data – model)	(130,518)	(-30,184)	(-16,505)	(-27,854)

This supplemental material has been provided by the authors to give readers additional information about their work.
